# Surgical Correction of Non-traumatic Patella Maltracking. Midterm Clinical Follow-up

**DOI:** 10.5041/RMMJ.10465

**Published:** 2022-04-26

**Authors:** Eran Keltz, Dror Ofir, Yiftah Beer, Naama Gruber, Mezen Falah, Gabriel Nierenberg

**Affiliations:** 1 Division of Orthopedic Surgery, Rambam Health Care Campus, Haifa, Israel; 2 The Ruth & Bruce Rappaport Faculty of Medicine, Technion–Israel Institute of Technology, Haifa, Israel; 3 Division of Orthopedic Surgery, Sourasky Medical Center, Tel Aviv, Israel; 4 Department of Orthopedic Surgery, Assaf Harofeh Medical Center, Zrifin, Israel; 5 Sports Traumatology & Cartilage Regeneration Service, Division of Orthopedic Surgery, Rambam Health Care Campus, Haifa, Israel

**Keywords:** Dislocation, Fulkerson, laxity, malalignment, maltracking, patella, realignment

## Abstract

**Background:**

Patellar instability comprises a group of pathologies that allow the patella to move out of its trajectory within the trochlear groove during walking. Symptomatic patients who need surgery commonly undergo soft tissue procedures such as medial patellofemoral ligament repair to strengthen the ligaments that hold the patella in place. However, soft-tissue repairs may be insufficient in patients suffering from patellar maltracking, which is characterized by an unbalanced gliding of the patella within its route. In these patients, a different approach is advised. We aim to provide the mid-term clinical outcomes of the Fulkerson distal realignment operation in selected patients with non-traumatic patellar maltracking.

**Methods:**

The clinical outcomes of the Fulkerson distal realignment operation performed in 22 knees of 21 patients were evaluated by a self-administered subjective International Knee Documentation Committee (IKDC) score and the Tegner–Lysholm knee scoring scale.

**Results:**

Before surgery, the median IKDC score was 52, and the median Tegner–Lysholm score was 56. Following surgery (mean follow-up 48 months, range 24–156), the median IKDC and the Tegner–Lysholm scores were 67 and 88, respectively. The improvement was statistically significant (*P*=0.001 and *P*=0.002 for IKDC and Tegner–Lysholm scores, respectively). Associated procedures included patella microfracture due to grade III–IV cartilage lesion (International Cartilage Repair Society grading system) in four patients, retinacular releases in three patients, medial capsular augmentations in two patients, and medial patellofemoral ligament reconstruction in two patients. One patient with Ehlers–Danlos disease required excessive medialization of the tibial tuberosity. Surgery-related complications occurred in three patients.

**Discussion:**

Surgical correction of patellar maltracking with Fulkerson distal realignment combined with associated procedures in individual patients was associated with an increase in subjective and functional clinical scores at medium-term follow-up. Particular attention should address pathologies associated with patellar maltracking and managed accordingly.

**Level of evidence:**

4c (case series).

## INTRODUCTION

Patellar instability is complex, with many clinical expressions ranging from painless grossly visible maltracking to painful chronic recurrent patellar dislocation/subluxation. Various parameters are taken into consideration when considering the different treatments options, such as the lower limb alignment (the hip-knee-ankle angle), static and dynamic patellar movements, and associated conditions such as connective tissue disorders.^1^,^2^

This study evaluated our experience with surgical treatment of patellar maltracking. Normally, the patella’s position is maintained within the trochlear groove when we ambulate. In patients with maltracking, flexion of the knee may lead the patella to move out of its course within the trochlear groove, either entirely (i.e. dislocation) or partially (i.e. subluxation). Maltracking, however, may not present with instability but with an unbalanced gliding of the patellar facets within the trochlea. Excessive pressure is applied, leading to patellofemoral pain and even osteoarthritis.^3^ Since various pathologies may allow maltracking, the subject of patellar maltracking and its proper treatment remains a challenge.^4^,^5^

A higher prevalence of patella dislocation is reported in young females, those with generalized ligamentous laxity, valgus knee, hip internal rotation, and increased quadriceps angle.^6^–^8^ Increased prevalence of valgus malalignment is common in individuals with high body mass index (BMI).^9^ It has been associated with young females engaged in dancing.^10^

If intra-articular loose bodies or chondral injury are ruled out, initial treatment is non-operative.^11^ Anti-inflammatory drugs, initial immobilization, and physiotherapy are the primary modalities in use. Surgical management is indicated in those with intra-articular loose bodies or a displaced osteochondral fracture. Surgery is also indicated in those with recurrent symptoms and those who engage in sporting activities.

Various surgical procedures have been described to correct the maltracking. Soft tissue procedures are aimed at strengthening the patella’s ligamentous attachments to keep it in place or restrain its pressure on the lateral facets. There are osseous procedures that alter the insertion point of the patellar tendon in order to change the vector of the forces employed on the patella during movement. The Fulkerson osteotomy is one such procedure, which is being evaluated in this study. The tibial tubercle, to which the tendon is attached, is detached from the tibia and reattached to the long bone medially and anteriorly,^12^ altering the quadriceps contraction force vector, and subtracting the applied pressure on the patellar facets. If the isolated bony procedure is insufficient, complementary retinacular, capsular, and cartilage repair modalities are added.^13^,^14^ A “wake test” with intraoperative active knee range of motion to monitor the accurate degree of correction is of marginal benefit.^1^ Evaluation by passive motion and gradual “on-demand” tubercle medialization prevails. The aim of this study was to prospectively evaluate the clinical outcomes of the Fulkerson realignment procedure in patients with demonstrable non-traumatic patella maltracking.

## METHODS

We prospectively followed a cohort of patients with clinically demonstrable patellar maltracking. The study was approved by the institutional research ethics committee. All patients underwent preoperative imaging that included full-length standing X-rays of the lower limbs to evaluate the hip-knee-ankle (HKA) angle for the degree of the valgus, lateral view of knee, and “sunrise” axial view of the patellofemoral joint in 45 degrees of knee flexion.

Patients were offered surgery if symptoms of maltracking did not improve following at least two physiotherapy courses. Patients were included if they suffered from painful recurrent patellar subluxation, or constant subluxation with crepitus and associated laxity (a minimum Beighton score^15^ of 3). Patients were also included if they suffered from a visible patella maltracking with recurrent effusion, or subluxation following previous soft tissue surgery that failed to correct the pathology.

Patients were excluded if the patellar instability was secondary to direct trauma or they had previously undergone knee surgery other than soft tissue repairs for maltracking. Patients were also excluded if their knee pain was due to other pathologies such as malunion around the knee, internal knee derangement (IKD; loose body, meniscal tears), calcifications at the tibial tuberosity (Osgood–Schlatter), and inflammatory joint disease.

The surgical approach consisted of a longitudinal midline incision, oblique biplanar osteotomy with complete detachment of a large tibia-based tuberosity fragment. Extending the lateral retinacula incision enabled patellar and trochlea cartilage inspection, evaluation of the entire extensor hood, and patellar response to trail passive range of motion while adapting the tubercle position to provide central isometric tracking of the patella. Extending the exposure to full lateral release was performed where residual tethering of lateral capsular scarring occurred. The extent of lateral retinacular release and the need for medial capsular plication were evaluated and completed according to the intraoperative passive range of motion test, demonstrating the new vector of patella glide and corrected tilt.

Clinical outcomes were evaluated by comparing the International Knee Documentation Committee (IKDC) score^16^ and the Tegner–Lysholm knee scoring scale^17^ before and after the operation. Both scores are patient-reported outcome scores that evaluate specific knee symptoms (swelling, clicking, stability) and function (walking, squatting, climbing stairs, etc.), with a range of 0–100 points (a higher score indicates a higher function). The scores were assumed to distribute non-normally, and non-parametric tests were used to evaluate differences observed for the IKDC and Tegner–Lysholm knee scores.

## RESULTS

**Figure 1:**
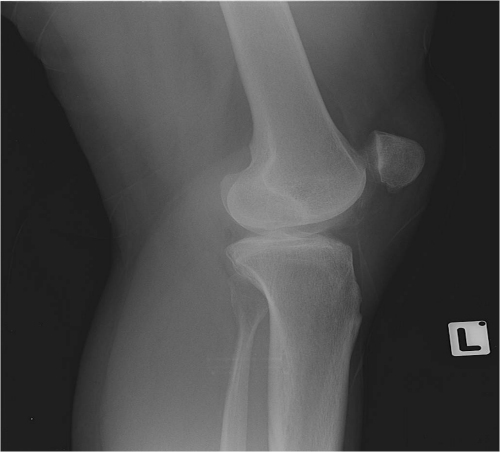
Lateral View in a Patient Diagnosed with Ehler-Danlos Demonstrating Patella Alta.

**Figure 2:**
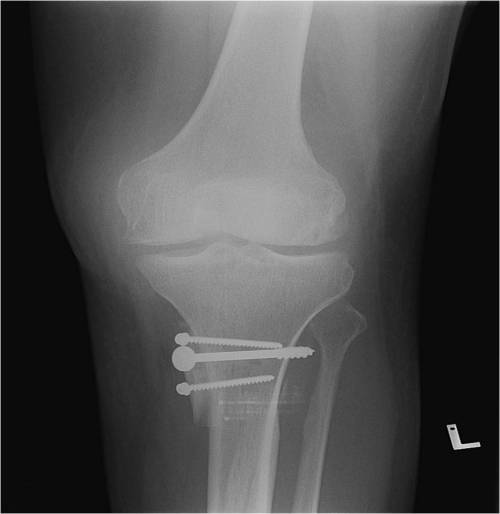
Postoperative True Anterior Posterior (AP) View of the Ehler-Danlos Patient with Far Medialization of the Tibial Tuberosity.

**Figure 3:**
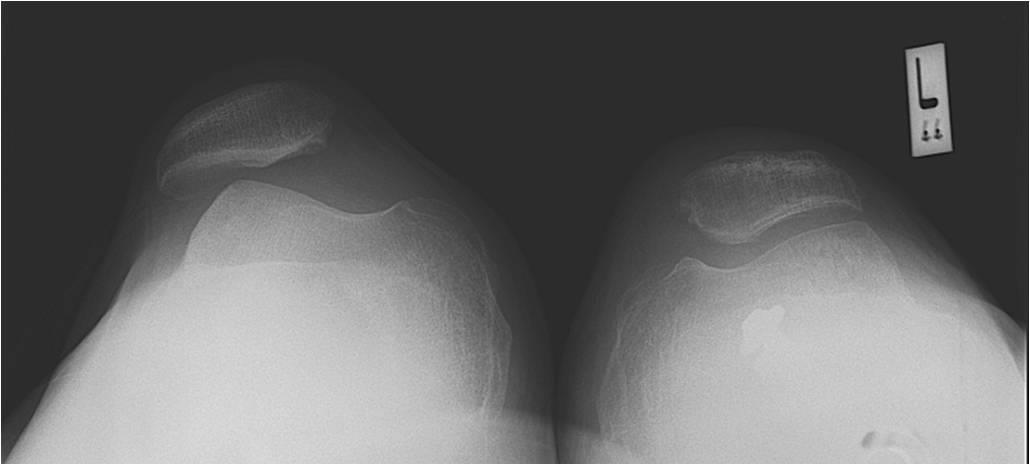
Postoperative Skyline View of Patella Demonstrating the Index Knee Compared to the Unoperated Contralateral Patella Position.

Included in this study were 21 patients in whom 22 knees underwent Fulkerson osteotomy of the tibial tuberosity for patellar maltracking (Table 1). Thirteen were females, and eight were males. The median age at surgery was 21.0 years (range 13–36). Seventeen patients suffered from constitutional ligamentous laxity. One patient had pathological laxity due to Ehler–Danlos syndrome (see Figures 1–3). One other patient had a diagnosis of rickets. Overall, 12 patients had genu valgum. None of the patients presented with varus alignment of the lower limbs. The median HKA angle was 186 degrees (range 182–191 degrees).

**Table 1:** Fulkerson Distal Realignment Patients: Demographics and Associated Surgical Procedures.

Pt	Age	Background Illness	Cartilage Debridement and Microfracture	Lateral Release	Medial Plication	Other Procedures
1	19	Ligamentous laxity	+			
2	34	Ligamentous laxity, atopic dermatitis	+			Autologous bone graft
3	36		+	+		
4	21	Ligamentous laxity			+	Autologous bone graft
5	31	Ehlers-Danlos, (ligamentous laxity)		+	+	Tibial tuberosity transfer
6	21	Ligamentous laxity		+		
7	22	Ligamentous laxity				
8*	20	Ligamentous laxity				Autologous bone graft
9	21			+	+	Autologous bone graft
10	28	Rickets; status post-MPFL reconstruction				
11	18	Ligamentous laxity	+	+	+	
12	13	Ligamentous laxity		+	+	
13	27	Ligamentous laxity		+	+	
14	18	Ligamentous laxity				
15	24					Revision Fulkerson
16	32	Ligamentous laxity				
17	26	Ligamentous laxity				
18	18	Ligamentous laxity				MPFL reconstruction†
19	48	Ligamentous laxity				MPFL reconstruction†
20	21	Ligamentous laxity				
21	20	Ligamentous laxity				

* Bilateral knee procedure.

† Autologous gracilis tendon.

MPFL, medial patellofemoral ligament; Pt, patient.

All patients underwent Fulkerson distal realignment osteotomy as a primary procedure. In eight patients, osteotomy was combined with a soft tissue procedure, i.e. a lateral release or a medial plication, or both. Two other patients had undergone previous medial patellofemoral ligament (MPFL) reconstruction. One patient had a consecutive bilateral correction. One patient diagnosed with Ehlers–Danlos syndrome suffered intractable bilateral chronic debilitating patellar instability. Centralizing the patella necessitated an extreme medial positioning of the tibial tuberosity (Figure 2).

Eight patients were found to have cartilage defects on the patella. In four of these, the cartilage defect extended beyond 50% of the cartilage depth (grade III according to the International Cartilage Repair Society grading system). The treatment of the cartilage lesions consisted of debridement and microfracture.

The mean follow-up was 48 months (range 24–156 months). The median IKDC score significantly improved from 52 (interquartile range [IQR] 39–71) before surgery to 66 (IQR 64–97) at final follow-up (*P*=0.001). The Tegner–Lysholm scores significantly improved from a median of 56 (IQR 44–77) to 88 (IQR 84–100) at the final follow-up (*P*=0.002).

One patient who suffered from a superficial surgical wound infection underwent a second surgery for the removal of screws. Four asymptomatic patients maintained a position of patella alta on postoperative imaging. One patient, a heavy smoker and highly atopic with a lengthy history of steroid treatments, had a delayed union that eventually resolved uneventfully.

All patients had a plain radiograph imaging demonstrating an uneventful union at the osteotomy site, and a sky-line view X-ray demonstrating centralized patella with no residual subluxation. No recurrence of patellar instability symptoms or mechanical disturbances was reported.

## DISCUSSION

**Figure 4:**
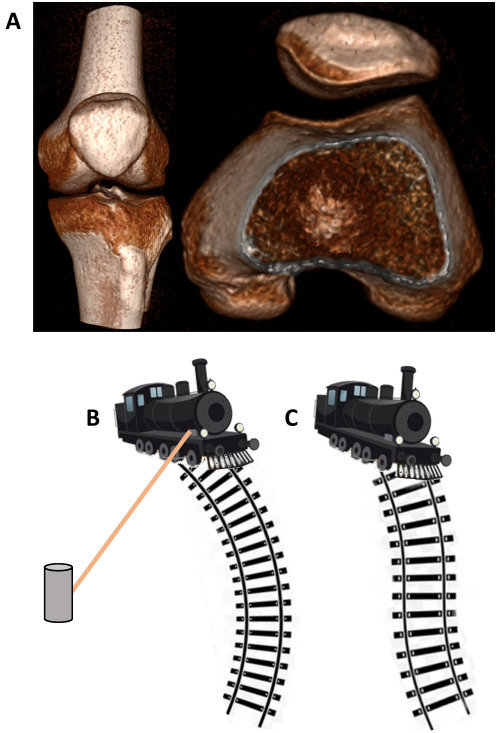
Treatment Concepts of Patellar Maltracking. **A:** The patellofemoral joint comprises a convex patella gliding upon a concave trochlea. This could be compared to a train struggling to remain on track. **B:** Medial patellofemoral ligament reconstruction is comparable to a tether stabilizing the train. **C:** However, the Fulkerson realignment procedure is comparable to altering the track itself.

The patellofemoral joint forms part of the knee joint. The patella is a sesamoid bone located within the quadriceps tendon. It protects the tendon and helps transform the force created by contraction of the quadriceps muscle contraction into knee extension, by increasing its working moment upon the proximal tibia. Proper gliding of the patella within the trochlear groove is essential for this function. Proper gliding is dependent on various factors, making pathology a multifactorial disturbance. It is dependent on trochlear morphology (trochlear sulcus angle, depth, and inclination), patellar facet morphology, and the axis and rotation of the lower limb (influenced by hip anteversion, tibial torsion, and knee alignment—genu valgum, for example) determining the forces employed by the surrounding soft tissue structures on the patella (Figure 4). Obviously, the quality of soft tissue itself has a detrimental influence on this complex motion, causing hyperlaxity and related syndromes (e.g. Ehler–Danlos, Marfan, etc.) to play a role.

Patients suffering from patellar instability, chronic subluxation, or recurrent dislocations commonly suffer from pain and mechanical knee discomfort. Physical findings such as crepitus, intermittent effusion, and functional disability are adverse prognostic factors that may render surgical treatment necessary.^18^ Whenever surgery is considered, it is essential to distinguish the “dislocator” from the “maltracker” since stabilization may be all that is necessary for the former,^19^ whereas a realignment procedure is crucial in the latter in order to alter the vector of forces employed on the patella during contraction of the quadriceps muscles.^3^ Reconstruction of medial restrains in patients with non-traumatic maltraction will often result in failure.^20^

The multiple pathologies treated by the Fulkerson distal realignment osteotomy and the multiplicity of surgical methods that have been employed on patients with maltracking make it difficult to evaluate the clinical results of different procedures in our patient population. The current literature does not always differ between those with recurrent dislocation and those with symptoms of maltracking, whether they have some degree of patellar subluxation or not (Figure 4).^21^ Palmer et al., for example, describe 84 knees reconstructed for severe patellofemoral maltracking, with 55% having frank dislocation and 45% with no instability.^22^ Pritsch et al. report tibial tubercle transfer in 80 knees in which 21.3% had no dislocation or subluxation.^23^ Hence, the variability between the patients treated makes it challenging to interpret the osteotomy results. On the other hand, relying on isolated soft tissue procedures like medial plication in cases of patellar instability has not demonstrated universal efficacy, with 22% recurrences of dislocation as reported by Efe et al.^24^

In our experience, extended surgical exposure is necessary for evaluating the general dynamics of the “extensor hood” and direct inspection of the patellofemoral gross anatomy and cartilage integrity. The exposure allows evaluating the added value of soft tissue procedures such as lateral release and medial plication. The lack of traditional imaging may be a drawback of this paper, not having measured parameters such as the tibial tuberosity to trochlear groove (TTTG) interval.^25^

Considering the large number of factors influencing maltracking, it is not surprising that there is insufficient evidence from the results of the surgical treatment of recurrent non-traumatic patella dislocation.^26^ In view of the natural history of the disease, where early degenerative changes may occur in the patellofemoral joint, we recommend addressing patellofemoral symptomatic maltracking and instability as early as possible.^14^,^27^

## Abbreviations

BMI: body mass index
HKA: hip-knee-ankle
IKD: internal knee derangement
IKDC: International Knee Documentation Committee
MPFL: medial patellofemoral ligament
TTTG: tibial tuberosity to trochlear groove.
